# Improving the Catalytic Activity of Hyperthermophilic *Pyrococcus horikoshii* Prolidase for Detoxification of Organophosphorus Nerve Agents over a Broad Range of Temperatures

**DOI:** 10.1155/2011/565127

**Published:** 2011-11-28

**Authors:** Casey M. Theriot, Rebecca L. Semcer, Saumil S. Shah, Amy M. Grunden

**Affiliations:** ^1^Division of Infectious Diseases, Department of Internal Medicine, The University of Michigan, Ann Arbor, MI 48109, USA; ^2^Department of Microbiology, North Carolina State University, Campus Box 7615, Raleigh, NC 27695-7615, USA; ^3^Biochemistry Branch, US Army Edgewood Chemical Biological Center, E3150 North Kingscreek Road, Aberdeen Proving Ground, Aberdeen, MD 21010-5183, USA

## Abstract

Prolidases hydrolyze Xaa-Pro dipeptides and can also cleave the P-F and P-O bonds found in organophosphorus (OP) compounds, including the nerve agents soman and sarin. *Ph1*prol (PH0974) has previously been isolated and characterized from *Pyrococcus horikoshii* and was shown to have higher catalytic activity over a broader pH range, higher affinity for metal, and increased thermostability compared to *P. furiosus* prolidase, *Pf*prol (PF1343). To obtain a better enzyme for OP nerve agent decontamination and to investigate the structural factors that may influence protein thermostability and thermoactivity, randomly mutated *Ph1*prol enzymes were prepared. Four *Ph1*prol mutants (A195T/G306S-, Y301C/K342N-, E127G/E252D-, and E36V-*Ph1*prol) were isolated which had greater thermostability and improved activity over a broader range of temperatures against Xaa-Pro dipeptides and OP nerve agents compared to wild type *Pyrococcus* prolidases.

## 1. Introduction

*Pyrococcus horikoshii *and *Pyrococcus furiosus* are both hyperthermophilic archaea, growing optimally at 98 –100°C that were isolated from a deep hydrothermal vent in the Okinawa Trough in the northeastern Pacific Ocean and from a shallow marine solfatara at Vulcano Island off the coast of Italy,respectively [1, 2]. *Pyrococcus *spp. are some of the most studied hyperthermophilic archaea to date owing in part to their utility for a variety of biotechnological applications [3–7]. For example, recombinant prolidases from *Pyrococcus *spp. are being studied for their potential use in bio-decontamination applications [8]. 

Prolidases function *in vivo* to hydrolyze dipeptides with proline in the C-terminus, Xaa-Pro, and a non-polar amino acid in the N-terminus [9]. However, studies have demonstrated that prolidases can also hydrolyze and detoxify organophosphate (OP) compounds such as chemical warfare agents (CWA) [8]. Two enzymes that have been characterized for potential field detoxification of nerve agents are organophosphorus acid anhydrolase (OPAA) and phosphotriesterase (PTE) [10, 11]. Recently, the crystal structure of OPAA from *Alteromonas* sp. JD6.5 strain has been solved, and it has been determined to be a prolidase [12]. While OPAA does have the capability to degrade OP nerve agents, its activity can be limited by exposure to high temperatures and solvents during use in field situations [13, 14].

In 2008, the Defense Threat Reduction Agency (DTRA), under the auspices of the Department of Defense recognized the importance of developing enzyme-based OP nerve agent detoxification systems and created an initiative calling for new enzymes and biocatalysts that are stable over a broad temperature and pH range, in the presence of salts and surfactants, and that do not pose an environmental hazard [15]. In response to the need to develop stable OP nerve agent degrading enzyme systems, thermostable prolidases from *Pyrococcus *spp. were studied [16–18].

Specifically, *Pyrococcus* prolidases from *P. furiosus* (PF1343 or *Pf*prol) and *P. horikoshii* (PH1149 or *Ph*prol and PH0974 or *Ph1*prol) were characterized both structurally and enzymatically [16, 18, 19]. The *Pyrococcus *prolidases were determined to be very similar, with *Pf*prol showing 88% amino acid similarity to *Ph*prol and 55% similarity to *Ph1*prol. Although they have high similarity to each other, the kinetic properties of *Ph1*prol appeared to be more favorable for application purposes than those of *Pf*prol and *Ph*prol. *Ph1*prol has demonstrated higher activity at lower temperatures and over a broader pH range. It has long-term stability, higher affinity for substrates, and a lower metal requirement for catalysis [16]. Therefore, it was deemed to be advantageous to use *Ph1*prol in mutagenesis studies in order to develop a better enzyme for OP nerve agent detoxification and to further investigate factors that influence the catalysis of thermophilic metalloenzymes. To this end, a random mutagenesis *Ph1*prol gene library was constructed and screened for production of mutants that showed increased prolidase activity at 30°C compared to wild type *Ph1*prol. Four *Ph1*prol mutants were isolated and subsequently characterized to determine their substrate catalysis over a broad range of temperatures and their performance against OP nerve agent analogs in comparison to *Ph1*prol and the previously characterized *Pf*prol.

## 2. Materials and Methods

### 2.1. Bacterial Strains, Media, and Materials

The *E. coli* K-12 derivative NK5525 (*proA*::Tn*10*) was used to construct the selection strain JD1(*λ*DE3) as described in [20] for screening of cold-adapted *P. horikoshii *prolidase variants. The *P. horikoshii* prolidase expression plasmid pET-*Ph1*prol was previously constructed as described in [16]. The *E. coli *strains were cultured either in Luria-Bertani (LB) broth or M9 selective minimal medium supplemented with 0.2% glucose, 1 mM MgSO4, 0.05% VitB_1_, 20 *μ*M IPTG, 20 *μ*M Leu-Pro. Ampicillin (100 *μ*g/mL), kanamycin (50 *μ*g/mL), chloramphenicol (34 *μ*g/mL), and tetracycline (6 *μ*g/mL) were added into the medium when required.

### 2.2. Construction of a Pool of pET-Ph1prol Plasmids Encoding Randomly Mutated *P. horikoshii* Prolidase Genes

Error-prone PCR mutagenesis using the Genemorph II Random mutagenesis kit (Stratagene, La Jolla, Calif) was used to amplify and insert mutations into the *P. horikoshii* prolidase gene (PH0974). PCR amplification was carried out for 30 cycles: (60 sec at 95°C, 60 sec at 55°C, 120 sec at 72°C), with a 10-minute final extension at 72°C. Reactions contained Mutazyme II reaction buffer, 125 ng/*μ*L of each primer, 40 mM dNTP mix, and 2.5 U of Mutazyme II DNA polymerase. Initial DNA template amounts used were 250 ng and 750 ng in order to select for medium-to-low mutation rates, respectively. The Genemorph II EZClone (Stratagene, La Jolla, Calif) reaction was employed to clone the mutated prolidase gene into the expression vector pET-21b. 

The EZClone reaction included EZClone enzyme mix, 50 ng of template plasmid (pET-prol), 500 ng of megaprimer (mutated prolidase PCR product), and EZClone solution. The reactions were amplified for 25 cycles: (50 sec at 95°C, 50 sec at 60°C, 14 min at 68°C). Amplified products were digested with *Dpn I* for 2 hours at 37°C to remove template plasmid. XL1-Gold super competent *E. coli* cells were transformed with the mutant plasmid mixture.

### 2.3. Screening for Increased Activity at Low Temperature

pET-*Ph1*prol plasmids from the mutant *P. horikoshii* library were transformed into the selective strain JD1(*λ*DE3) and were plated on M9 selective agar plates. Colonies that grew after being incubated for 3 –7 d at 20°C were isolated on LB plates and then grown in 10 mL LB medium at 37°C with shaking (200 rpm) until an optical density of 0.6–0.8 was reached. IPTG was then added to the cell culture to a final concentration of 1 mM. The induced culture was shaken at 37°C for 3 h before harvesting the cells. These cell pellets were lysed using 300 *μ*L of B-per buffer (Thermo Scientific, Rockford, Ill), and the resulting cell extracts were used for enzyme activity assays conducted at 30°C and at 100°C. Heat-treated soluble protein samples were heated at 80°C for 20 min. Four mutant colonies that exhibited at least 2-3-fold higher activities compared to the cells expressing the wild type *P. horikoshii *prolidase were selected for characterization, and their plasmids were isolated. The prolidase genes present in those isolated plasmids were sequenced using the T7 promoter and T7 terminator primers (MWG Biotech, Huntsville, AL).

### 2.4. Large-Scale Expression of Recombinant *P. horikoshii* Prolidase Mutants

Production of *P. horikoshii *prolidase variants (A195T/G306S-, Y301C/K342N-, E127G/E252D-, and E36V-*Ph1*prol) was carried out in transformed *E. coli *BL21 (*λ*DE3) cells with the appropriate pET-*Ph1*prol plasmid and pRIL vector. The transformants were grown in 1 L cultures of autoinduction media [21] incubated at 37°C with shaking (200 RPM) overnight.

### 2.5. Purification of Recombinant *P. horikoshii* Prolidase Mutants

Cell pellets from the four *Ph1*prol variants (A195T/G306S-, Y301C/K342N-, E127G/E252D-, and E36V-*Ph1*prol) were suspended in 50 mM Tris-HCl, pH 8.0 (3 mL Tris per 1 gram of cell paste), with 1 mM benzamidine and 1 mM DTT. For each variant, diluted cell slurry was passed through a French pressure cell (20,000 lb/in^2^) three times. Cell lysates were centrifuged at 38,720 x g for 30 min at 4°C, and then the supernatants were heated to 80°C for 30 min anaerobically to denature any proteins not stable at that temperature. Heat-treated supernatants were centrifuged at 38,720 x g to remove the denatured proteins. Supernatants were sampled both before and after heat treatment for activity analysis. (NH_4_)_2_SO_4_ was added gradually to the supernatants to make a final concentration of 1.5  M prior to loading onto a 5 mL Phenyl-Sepharose hydrophobic interaction chromatography column (Hi-Trap Phenyl HP Column, GE Healthcare Life Sciences, Piscataway, NJ). Fractions containing the prolidase mutants were pooled and dialyzed overnight into 4 L of 50 mM Tris HCl, pH 8.0 at 4°C, and were further purified on a 5 mL Q Sepharose anion exchange chromatography column (Hi-Trap Q FF Column, GE Healthcare Life Sciences, Piscataway, NJ). Buffers for both purification steps have been described in [16]. Fractions from both purification steps were further visualized using SDS-PAGE (12.5% SDS-polyacrylamide gels) and were tested for enzyme activity. Fractions were then pooled based on gel images, and enzyme stocks were stored at −80°C. The molecular weights of *Ph1*prol and mutants are approximately 40.04 kDa. The purity of each *Ph1*prol mutant was estimated to be greater than 95% using both visual inspection of SDS-polyacrylamide gels and electrophoretic microchip analysis.

### 2.6. Prolidase Enzyme Activity Assay

The enzyme activity assay protocol is based on a method previously described by [17, 20]. The reaction mixture (500 *μ*L) contained 50 mM MOPS buffer (3-[*N*-morpholino] propanesulfonic acid) pH 7.0, 200 mM NaCl, water, 5% (vol/vol) glycerol, 100 *μ*g/mL BSA (bovine serum albumin) protein, 0.2 mM CoCl_2_, and the enzyme. The reaction mixture was heated at 100°C for 5 min allowing time for the metal and enzyme to interact. The reaction was initiated by the addition of substrate (Xaa-Pro, 4 mM final concentration) and allowed to proceed for 10 min at 100°C. The reaction was stopped with 500 *μ*L glacial acetic acid and 500 *μ*L ninhydrin reagent (3% (wt. vol)) and heated again for 10 min at 100°C. The reaction was then cooled to 23°C. Prolidase samples were assayed in triplicate and specific activity was calculated using the absorbance value at 515 nm and an extinction coefficient of 4,570 M^-1 ^cm^−1^ for the ninhydrin-proline complex.

For assays evaluating the temperature profile, WT-*Ph1*prol and the four prolidase mutants were assayed in triplicate for activity with 4 mM Met-Pro at 10°C, 20°C, 35°C, 50°C, 70°C, and 100°C. Experiments were performed in duplicate. 

### 2.7. Substrate Specificity and Kinetics Experiments

In order to study substrate specificity, the following Xaa-Pro dipeptides were used as substrates (4 mM final concentration) in the enzyme activity assays for WT-*Ph1*prol and the four prolidase mutants: Val-Pro, Met-Pro, Phe-Pro, Leu-Pro, Ala-Pro, and Gly-Pro. Prolidase samples were assayed with two additional substrates, Pro-Ala and Val-Leu-Pro, to further illustrate prolidase preference of Xaa-Pro dipeptides [20]. Kinetic parameters of the *Ph1*prol mutants were determined at 70°C using a range of Leu-Pro concentrations (0.25–12 mM).

### 2.8. Thermostability and Pot-Life Experiments

Thermostability experiments were performed in duplicate on WT-*Ph1*prol and the four mutants. Each enzyme was diluted to a concentration of 0.04 mg/mL in 50 mM MOPS, pH 7.0, and 200 mM NaCl and incubated in an anaerobic sealed vial at 90°C. An initial sample was taken to represent Time = 0 h, and additional samples were taken at Time = 24, 48, and 72 h. Samples were diluted to 0.4 *μ*g/mL in 50 mM MOPS, pH 7.0, and 200 mM NaCl and were then assayed in triplicate in accordance with the enzyme activity assay protocol described in Section 2.6. In all cases, the substrate used in the activity assay was 4 mM Met-Pro. 

Pot-life experiments were also performed in duplicate. Each enzyme was diluted to a concentration of 0.04 mg/mL in 50 mM MOPS, pH 7.0, and 200 mM NaCl and incubated anaerobically in a sealed vial at 70°C. An initial sample was taken to represent Time = 0 days, and additional samples were taken at Time = 1, 7, 14, 16, and 21 days. Samples were diluted to 0.4 *μ*g/mL in 50 mM MOPS, pH 7.0, and 200 mM NaCl and were then assayed in triplicate to determine specific activity. 

### 2.9. DSC Experiment

Differential scanning calorimetry was performed using a MicroCal VP-DSC scanning calorimeter. The calorimetric samples contained ~1 mg/mL protein in 50 mM MOPS, 200 mM NaCl, 0.2 mM CoCl_2_, pH 7.0. Protein samples were dialyzed 15 h against this buffer before the experiment. Samples were degassed before loading into the chamber cell. The calorimetric experiment was performed by heating the samples at a scan rate of 100°C/hr.

### 2.10. DFP (diisopropylfluorophosphate) Assay

The hydrolysis of DFP by prolidases was measured by monitoring fluoride release with a fluoride-specific electrode as previously described [22]. Assays were performed at 35°C and 50°C, with continuous stirring in 2.5 mL of buffer (50 mM MOPS, 200 mM NaCl, pH 7.0), 0.2 mM CoCl_2_ and 3 mM DFP. The enzyme and metal were incubated at the reaction temperature 5 min prior to the start of the reaction. The background of DFP hydrolysis was measured by running a reaction without enzyme present at 35°C and 50°C. The background hydrolysis of DFP was subtracted from enzymatic hydrolysis to determine specific activity of the enzyme.

### 2.11. *p*-Nitrophenyl Soman Assay (O-Pinacolyl *p*-Nitrophenyl Methylphosphonate Activity)

Prolidase hydrolysis of *p*-nitrophenyl soman was monitored by accumulation of *p*-nitrophenolate [10, 22]. The p-Nitrophenyl Soman was synthesized at Edgewood Chemical Biological Center in Aberdeen Proving Ground, Md. and contained a racemic mixture of all four stereoisomers. The purity of the soman analog was greater than 90% based on gas chromatography analysis [12]. Two mL reaction assays contained buffer (50 mM MOPS, 200 mM NaCl, pH 7.0), 0.2 mM CoCl_2_, and 0.3 mM *p*-nitrophenyl soman. The reactions were conducted at three different temperatures (35*°*C, 50*°*C and 70*°*C). The enzyme and metal were incubated at the specified reaction temperature 5 min prior to the start of the reaction. Absorbance of the product *p*-nitrophenolate was measured at 405 nm over a 5 min range.To calculate activity, the extinction coefficient for *p*-nitrophenolate of 10,101 M^−1^ cm^−1^ was used.

## 3. Results and Discussion

### 3.1. *P. furiosus* and *P. horikoshii* Prolidase-Specific Activities with OP Nerve Agents: DFP and Soman Analog

**Figure 1:**
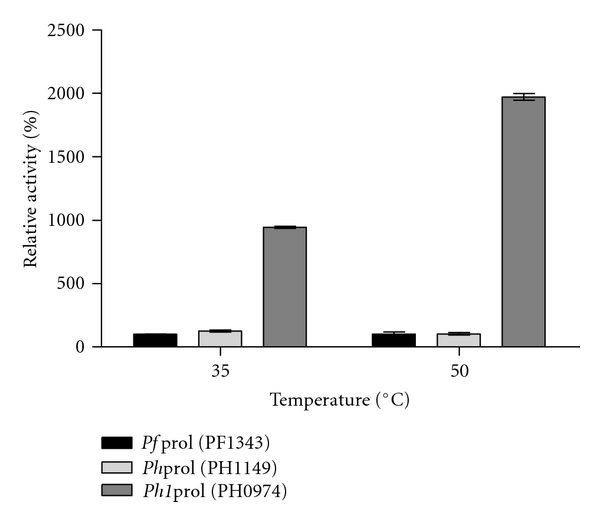
Relative activity with WT-*Pf*prol and *P. horikoshii* prolidases with OP nerve agent DFP. All prolidase assays contained 50 mM MOPS, 200 mM NaCl, pH 7.0, 0.2 mM CoCl_2_, and 3 mM DFP. 100% relative activity corresponds to WT-*Pf*prol specific activity for DFP of 0.42 *μ*moles product formed/min/mg at 35*°*C and 0.73 *μ*moles product formed/min/mg at 50*°*C.

**Figure 2:**
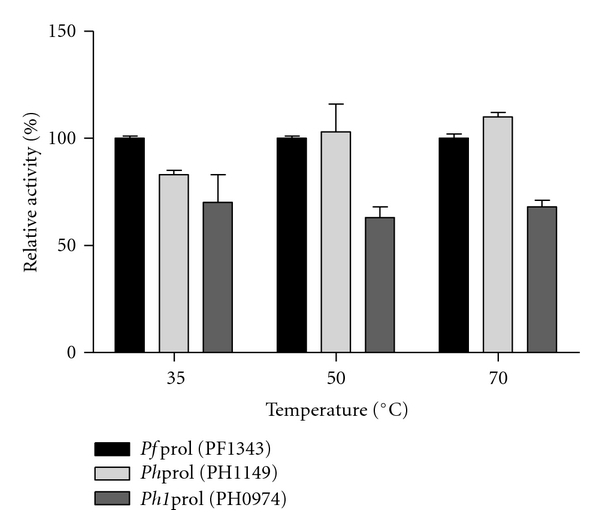
Relative activity with WT-*Pf*prol and *P. horikoshii *prolidase with OP nerve agent analog, *p*-nitrophenyl soman. All prolidase assays contained 50 mM MOPS 200 mM NaCl pH 7.0, 0.2 mM CoCl_2_, and 3 mM *p*-nitrophenyl soman. One hundred percent relative activity corresponds to WT-*Pf*prol-specific activity for *p*-nitrophenyl soman of 0.26 *μ*mol product formed/min/mg at 35°C, 0.33 *μ*mol product formed/min/mg at 50°C, and 0.56 *μ*mol product formed/min/mg at 70°C.

Previous studies characterizing *P. horikoshii *prolidase homolog 1 (*Ph1*prol, PH0974) demonstrated that it has higher catalytic activity over a broader pH range, higher affinity for metal, and is more thermostable than either *P. furiosus *prolidase (*Pf*prol, PF1343) or *P. horikoshii *prolidase (*Ph*prol, PH1149) when assayed with the dipeptide substrate Met-Pro [20]. Based on these favorable attributes for *Ph1*prol when reacting with its natural substrates, Xaa-Pro dipeptides, there was interest in determining the relative activity of recombinant *Ph1*prol compared to *Pf*prol and *Ph*prol against G-type nerve agent simulants DFP and soman analog, *p*-nitrophenylsoman. As indicated in Figure 1, DFP exhibited the greatest hydrolysis with *Ph1*prol. *Ph1*prol had a relative activity that was 843% higher than *Pf*prol and 817% higher than *Ph*prol at 35°C and 1870% higher than both *Pf*prol and *Ph*prol at 50°C (Figure 1). In contrast, the trends with the soman analog were very different as shown in Figure 2. The relative activity of *Ph1*prol was only 70%, 63%, and 68% of the  *Pf*prol activity at 35°C, 50°C, and 70°C, respectively (Figure 2). These results indicate that *Ph1*prol has a preference for DFP and does not exhibit high activity with the soman analog. Differences in the protein structures likely play a role in the substrate preference since *Pf*prol and *Ph1*prol share only 55% amino acid residue similarity [16]. By altering the *Ph1*prol structure further using a random mutagenesis approach, it would be possible to isolate *Ph1*prol variants that show even greater hydrolysis of DFP and/or improved activity against the soman analog.

### 3.2. Screening and Isolation of the *Ph1*prol Mutant Population Using Proline Auxotrophic Strain JD1 (*λ*DE3)

Since *Ph1*prol showed the most favorable properties including higher activity with DFP, it was selected for further mutagenesis using an error-prone PCR strategy, which employs a mutated polymerase. Transformed *E. coli* JD1 (*λ*DE3) cells were used to select for *Ph1*prol variants on minimal media plates that were supplemented with 20 *μ*M Leu-Pro and grown at 20°C. Colonies that were visible in 3 –7 days were plated on minimal and rich (LB) media. *Ph1*prol variants were screened using small-scale expression cultures (10 mL) induced with IPTG. Four *Ph1*prol variants out of over 200 screened were selected for sequencing after showing two-fold higher activity with Leu-Pro at 30°C and somewhat reduced activity at 100°C as compared to wild type. The increased activity at the lower temperature of 30°C and variation at the higher temperature of 100°C is indicative of a mutation in a thermophilic protein, which can compromise activity or stability at higher temperatures but could create more flexibility and increased catalysis at the lower temperatures. 

### 3.3. Sequencing of *Ph1*prol Mutants

**Figure 3:**
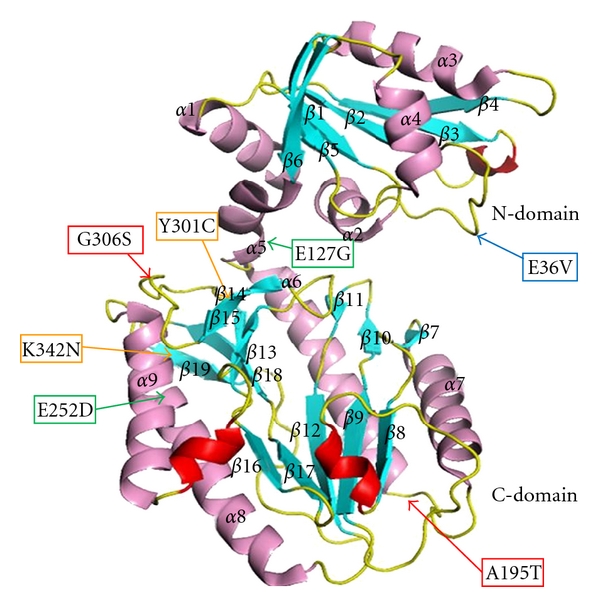
Mapping of the mutations in the monomeric structure of *P. horikoshii *prolidase (*Ph1*prol or PH0974). Mutations made in the *Ph1*prol are indicated by colored arrows where red indicates mutations present in Mutant no.10 (A195T/G306S-*Ph1*prol), orange indicates mutations in Mutant no.19 (Y301C/K342N), green indicates mutations in Mutant no.35 (E127G/E252D), and blue indicates mutations in Mutant no.72 (E36V). The domain structure of *Ph1*prol is presented as a ribbon drawing where the N-terminal (residues 1–120) and C-terminal (131–356) domains are labeled and are connected by a *α*-helical linker at residues 121–130. The putative active site pocket is located between two 3_10_ helixes (two red helices, residues 191–195 and 281–284) (modified from [18]).

Prior to sequencing, the four *Ph1*prol mutants were numbered (10, 19, 35, and 72) based solely on the order in which they had been isolated. Sequencing of the variants revealed the locations of the amino acid substitutions for each mutant (Figure 3). Mutant no.10 has two mutations: one at position 195 in which there is a change from alanine to threonine (A195T) and the second at amino acid residue 306 in which glycine is changed to serine (G306S). Both of these mutations reside in the C-terminal region of *Ph1*prol. Mutant no.19 has two mutations: one at position 301 in which there is a change from tyrosine to cysteine (Y301C) and the second at amino acid residue 342 which has a substitution of lysine with an asparagine (K342N). Both of these mutations are in the C-terminal region of the enzyme. Mutant no.35 contains two mutations: one at position 127 in the *α*-helical linker region in which there is a change from glutamate to glycine (E127G) and the second in the C-terminal region at position 252 with a substitution of glutamate for aspartate (E252D). Mutant no.72 is the only mutation in the N-terminal region at position 36 with a change from glutamate to valine (E36V) in *Ph1*prol. The mutations are remote from the active site pocket, which is shown in Figure 3 as being located between two 3_10_ helixes (red helices, residues 191–195, and 281–284). Therefore, the mutations are not likely directly changing the active site chemistry. Rather the mutations such as E36V, E127G, and Y301C may be affecting prolidase dimerization as those residues are located along the dimerization interface. Furthermore, the mutations may be affecting the conformational dynamics of the enzymes since some of the mutations are located in the loop and linker regions. The change in conformation dynamics is giving the variants better activity over a broader range of temperatures as indicated in Section 3.4.

### 3.4. Effects of Mutagenesis on the Temperature Profile of *P. horikoshii* Prolidase Variants

**Figure 4:**
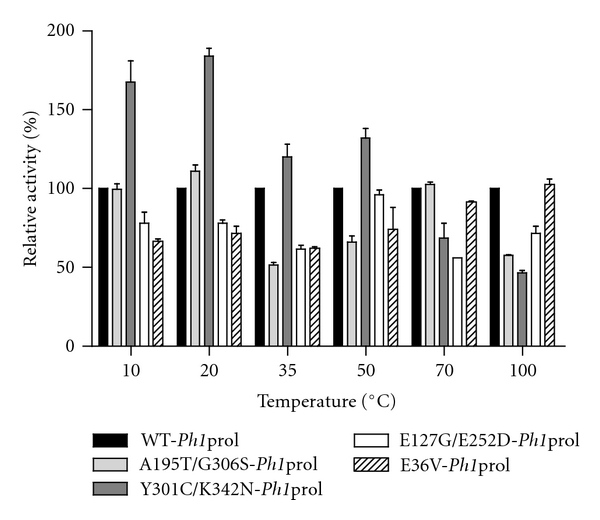
Temperature profile of WT-*Ph1*prol and the four variants. Relative activities are shown as a percentage of the WT-*Ph1*prol. Prolidase assays were performed in triplicate at 10°C, 20°C, 35°C, 50°C, 70°C, and 100°C and contained 0.2 mM CoCl_2_ and 4 mM Met-Pro. WT-*Ph1*prol-specific activity was 66 U/mg at 10°C, 131 U/mg at 20°C, 246 U/mg at 35°C, 340 U/mg at 50°C, 973 U/mg at 70°C, and 3,955 U/mg at 100°C.

Both the wild type *Ph1*prol and the four variants were most active at 100°C (Figure 4). WT-*Ph1*prol and E36V-*Ph1*prol had very high specific activities (3,955 U/mg and 4094 U/mg, resp.) with 4 mM Met-Pro, both of which are twice as high as that of *Pf*prol at 100°C (2,154 U/mg) [21]. A195T/G306S- and E127 G/E252D-*Ph1*prol were slightly less active (2,307 U/mg and 2,831 U/mg, resp.) than WT *Ph1*prol, and Y301C/K342N-*Ph1*prol showed the lowest level of activity in comparison to the other mutants at 1,842 U/mg. Activity was reduced by more than half at 70°C for all of the variants; however, WT-*Ph1*prol, A195T/G306S-, and E36V-*Ph1*prol all had specific activities close to 1,000 U/mg (973 U/mg, 1000 U/mg, and 913 U/mg, resp.), whereas the specific activity of *Pf*prol at 70°C was 806 U/mg [21]. At 50°C, Y301C/K342N-*Ph1*prol had a higher specific activity than any of the other variants and the wild type (450 U/mg) and was roughly three times more active than *Pf*prol at 50°C [21]. 

At the lower temperatures, 35°C, 20°C and 10°C, Y301C/K342N-*Ph1*prol out-performs the other *Ph1*prol variants, the WT-*Ph1*prol, the WT-*Pf*prol and R19G/G39E/K71E/S229T-*Pf*prol (the highest performing *Pf*prol mutant at lower temperatures) [21]. At 35°C, Y301C/K342N-*Ph1*prol (298 U/mg) has relative activity that is 121% that of WT-*Ph1*prol, 489% that of WT-*Pf*prol, and 244% that of R19G/G39E/K71E/S229T-*Pf*prol (246 U/mg, 61 U/mg, and 122 U/mg, resp.). At 20°C, Y301C/K342N-*Ph1*prol (241 U/mg) has a relative activity 184% that of WT-*Ph1*prol, 964% higher than WT-*Pf*prol, and 482% higher than R19G/G39E/K71E/S229T-*Pf*prol (specific activities of 131 U/mg for WT-*Ph1*prol, 25 U/mg for WT-*Pf*prol and 50 U/mg for R19G/G39E/K71E/S229T-*Pf*prol) [21]. The greatest improvement in performance for the *Ph1*prol mutants is seen when assayed at 10°C. Y301C/K342N-*Ph1*prol (109 U/mg) has a relative activity that is 166% higher than that of WT-*Ph1*prol, 1,982% higher than that of WT-*Pf*prol, and 396% higher than R19G/G39E/K71E/S229T-*Pf*prol (specific activities of 66 U/mg for WT-*Ph1*prol, 5.5 U/mg for WT-*Pf*prol, and 27.5 U/mg for R19G/G39E/K71E/S229T-*Pf*prol) [21].

### 3.5. Effects of Mutagenesis on Substrate Specificity and Kinetics of *P. horikoshii* Prolidase Variants

Substrate specificity of WT-*Ph1*prol is shown in Table 1 along with the specific activities of A195T/G306S-, Y301C/K342N-, E127G/E252D-, and E36V-*Ph1*prol, which are reported as a percentage relative to the activity of the wild type. WT-*Ph1*prol was most active with the dipeptide Val-Pro (4,602 U/mg), while mutants A195T/G306S-, Y301C/K342N-, E127G/E252D-, and E36V-*Ph1*prol had much lower activity at 33%, 36%, 46%, and 56% of the WT *Ph1*prol, respectively. A195T/G306S-*Ph1*prol showed the highest activity with Met-Pro at 143% that of the wild type (2,809 U/mg), while Y301C/K342N-, E127G/E252D-, and E36V-*Ph1*prol preferred Ala-Pro with specific activities of 183%, 324%, and 556% that of WT-*Ph1*prol (1,452 U/mg). WT-*Ph1*prol seems to prefer the most hydrophobic amino acids, while the four variants have the highest activity with a less hydrophobic amino acid in the N-terminal position of the dipeptide substrate. While alanine is considered to be a hydrophobic amino acid, it is less hydrophobic than valine, methionine,phenylalanine, and leucine and is most similar in structure to glycine. WT-*Ph1*prol has much lower activity with Gly-Pro than with Ala-Pro (369 compared to 1,452 U/mg). A195T/G306S-, Y301C/K342N-, and E127G/E252D-*Ph1*prol have less or similar activity with Gly-Pro as compared to WT-*Ph1*prol (369 U/mg) while E36V-*Ph1*prol has 444% higher activity. 

Specific activities were consistently low with both Pro-Ala and Val-Leu-Pro (2 U/mg, WT-*Ph1*prol) for the wild type *Ph1*prol and the four mutants. Due to the nature of the prolidase enzyme and its unique ability to cleave the bond between Xaa-Pro dipeptides, it was expected that the enzyme would not show any notable activity with a Pro-Xaa dipeptide or a tripeptide. While two of the four variants (mutants A195T/G306S- and E36V-*Ph1*prol) show increased activity with Pro-Ala when compared to wild type, it must be noted that specific activity with Pro-Ala for WT-*Ph1*prol was extremely low, at only 14 U/mg. Y301C/K342N-*Ph1*prol had 88% WT activity with Val-Leu-Pro; however, WT-*Ph1*prol-specific activity was only 2 U/mg. 

The catalytic activities of WT-*Ph1*prol and its mutants were tested at 70°C with Leu-Pro (Table 2). All *Ph1*prol mutants had higher *k*_cat_ values than the WT-*Ph1*prol suggesting that the amino acid changes in the mutant enzymes did have an effect on structure and ultimately the catalytic activity of the prolidase with the substrate Leu-Pro. Although the *k*_cat_ values were higher, the overall enzyme turnover rates were not for some of the mutants compared to WT-*Ph1*prol. All the *Ph1*prol mutants showed an increased turnover rate, *k*_cat_/*K*_*m*_, with Leu-Pro except for Y301C/K342N-*Ph1*prol. This could be due to the increase in the *K*_*m*_ of this mutant, which is almost three times higher than WT-*Ph1*prol.

### 3.6. Effects of Amino Acid Substitutions on the Thermostability and Pot-Life Activity of *P. horikoshii* Prolidase Mutants

To determine whether the amino acid substitutions in the four* Ph1*prol variants had any effect on thermostability, the mutants were incubated in sealed vials at 90°C, anaerobically, for 72 h. Samples were taken every 24 h to measure catalytic activity with Met-Pro (4 mM) as the substrate. In Theriot et al. 2010, it was shown that WT-*Pf*prol was more thermostable than its mutants and had lost 50% activity with Met-Pro by 21 hours at 90°C [20]. After 40 h at 90°C, WT-*Ph1*prol had lost 50% activity. Mutants Y301C/K342N-*Ph1*prol and E127G/E252D-*Ph1*prol both demonstrated increased thermostability at 90°C compared to wild type and had lost 50% activity after 58 hours of incubation. Mutants A195T/G306S-*Ph1*prol and E36V-*Ph1*prol displayed a 50% loss of activity after 32 and 35 hours at 90°C, respectively. Both WT-*Ph1*prol and the four *Ph1*prol mutants were stable at 90°C for a significantly longer time period than *Pf*prol and its mutants [20].

**Figure 5:**
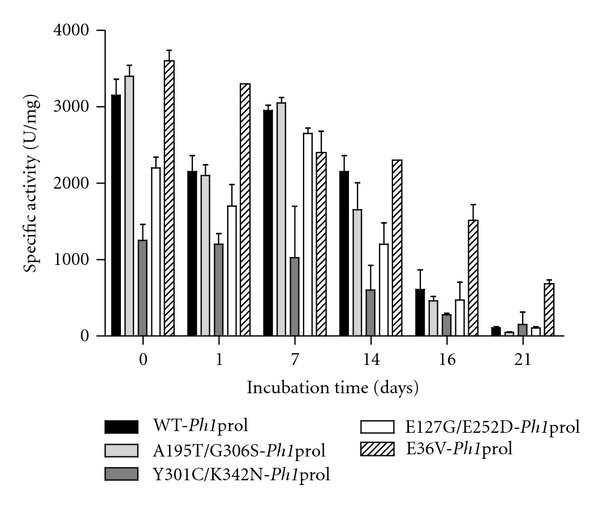
Pot-life activity of WT-*Ph1*prol and prolidase mutants incubated anaerobically at 70°C. Pot-life experiments were performed in duplicate and samples were taken at 0, 1, 7, 14, 16, and 21 days to assess reactivity. Prolidase assays were performed in triplicate at 100°C and contained 0.2 mM CoCl_2_ and 4 mM Met-Pro. Error bars in Figure 5 represent standard deviation of specific activity across duplicate runs.

Pot-life activity was monitored over the course of 21 days with samples taken every seven days until day 14 and then again on days 16 and 21 while the samples were incubated anaerobically at 70°C (Figure 5). Pot-life experiments with WT-*Pf*prol and its mutants as reported in Theriot et al., 2010 were conducted over a 48 h period at 70°C [20]. In preliminary pot-life experiments with WT-*Ph1*prol and its mutants, the specific activities with Met-Pro were still remarkably high after 48 h (no obvious decrease in specific activity was detected; data not shown), so the experiments were continued until the enzymes were considered to be no longer active (21 days). Initial specific activities for WT-*Ph1*prol, A195T/G306S-, Y301C/K342N-, E127 G/E252 D-, and E36V-*Ph1*prol were 3,150 U/mg, 3,400 U/mg, 1,250 U/mg, 2,200 U/mg, and 3,600 U/mg, respectively, with 4 mM Met-Pro as the substrate. WT-*Ph1*prol had lost 50% activity by day 12 of incubation at 70°C. Mutants Y301C/K342N-, E127G/E252D-, and E36V-*Ph1*prol had 50% activity remaining by days 12, 13, and 14, respectively. A195T/G306S-*Ph1*prol was at 50% activity after 10 days at 70°C. By 21 days at 70°C, all five prolidases were at or below 25% of the initial activity.

As reported in Theriot et al., 2010, the specific activities of WT-*Pf*prol and its three mutants (G39E-, R19G/K71E/S229T-, and R19G/G39E/K71E/S229T-*Pf*prol) were 1,083 U/mg, 599 U/mg, 722 U/mg, and 4,496 U/mg, respectively, with 4 mM Met-Pro after 48 h of incubation at 70°C [20]. In contrast, after 7 days (or 168 h) at 70°C, WT-*Ph1*prol, A195T/G306S-, Y301C/K342N-, E127G/E252D-, and E36V-*Ph1*prol had specific activities of 2,950 U/mg, 3,050 U/mg, 1,230 U/mg, 2,650 U/mg, and 2,400 U/mg, respectively, with 4 mM Met-Pro. After 48 h at 70°C, WT-*Pf*prol had a higher specific activity than any of its mutants (1,083 U/mg). After 7 days at 70°C, Y301C/K342N-*Ph1*prol showed the lowest specific activity of the *Ph1 *prolidases; however, it still had activity 114% that of WT-*Pf*prol at 48 h [20].

### 3.7. DSC Results

The thermal stability of wild type *Ph1*prol and variants was determined by differential scanning calorimetry (DSC) experiments.  Table 3 shows the denaturation temperature of the wild type and variant enzymes. The mutations that improved catalytic activity of the* Ph1*prol at lower temperatures did not adversely affect the temperature stability of the enzymes. 

### 3.8. Effects of Amino Acid Substitutions on Substrate Specificity with OP Nerve Agents DFP and Soman Analog, *p*-Nitrophenyl Soman

**Figure 6:**
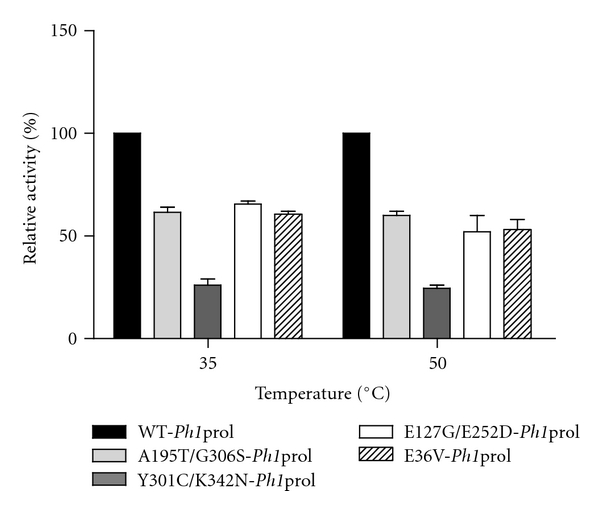
Relative activity with WT-*Ph1*prol and prolidase mutants with OP nerve agent DFP. All prolidase assays contained 50 mM MOPS 200 mM NaCl pH 7.0, 0.2 mM CoCl_2_, and 3 mM DFP. One hundred percent relative activity corresponds to WT-*Ph1*prol specific activity for DFP of 4 *μ*mol product formed per minute per milligram at 35°C and 10 *μ*mol of product formed per minute per milligram at 50°C.

Substrate specificity of WT-*Ph1*prol with DFP is shown in Figure 6 along with the specific activities of A195T/G306S-, Y301C/K342N-, E127G/E252D-, and E36V-*Ph1*prol, which are reported as a percentage relative to the activity of the wild type. WT-*Ph1*prol was most active with DFP at 35°C and 50°C with a specific activity of 4 U/mg and 10 U/mg, respectively. The mutants A195T/G306S, Y301C/K342N-, E127G/E252D-, and E36V-*Ph1*prol had significantly lower activity with DFP; even at 50°C the activity was only 59%, 25%, and 55% of WT-*Ph1*prol activity. However, it should be noted that the *Ph1*prol mutants have 808%, 183%, and 402% (A195T/G306S, Y301C/K342N-, E127G/E252D-, and E36V-*Ph1*prol, resp.) of the DFP activity compared to WT *P. furiosus *prolidase and also compared favorably to the highest DFP activity reported for the R19/G39E/K71E/S229T *Pf*prol mutant, which was shown to have 398% higher activity than WT *Pf*prol [21].

**Figure 7:**
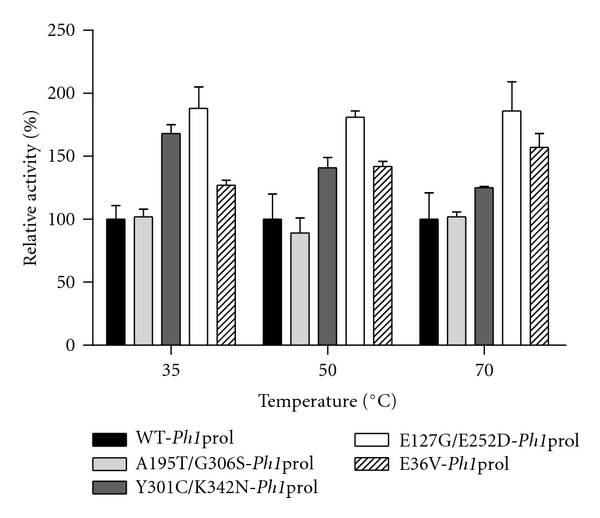
Relative activity with WT-*Ph1*prol and prolidase mutants with OP nerve agent analog, *p*-nitrophenyl soman. All prolidase assays contained 50 mM MOPS 200 mM NaCl pH 7.0, 0.2 mM CoCl_2_, and 3 mM *p*-nitrophenyl soman. One hundred percent relative activity corresponds to WT-*Ph1*prol specific activity for *p*-nitrophenyl soman of 0.26 *μ*mol product formed per minute per milligram at 35°C, 0.33 *μ*mol product formed per minute per milligram at 50°C, and 0.56 *μ*mol product formed per minute per milligram at 70°C.

Figure 7 reveals a different trend, where the mutations in WT-*Ph1*prol increased the specific activity with the soman analog, *p*-nitrophenyl soman. WT-*Ph1*prol showed the highest activity with the soman analog at 70°C, with a specific activity of 0.56 U/mg. The mutant A195T/G306S-*Ph1*prol had a similar specific activity to WT-*Ph1*prol when incubated at 35°C, 50°C, and 70°C. Mutants Y301C/K342N-, E127G/E252D-, and E36V-*Ph1*prol showed increased activity with the soman analog over WT-*Ph1*prol at each assay temperature. The most significant specific activities with the soman analog were seen with Y301C/K342N-, E127G/E252D-, and E36V-*Ph1*prol at 70°C, which correlated to 125%, 186%, and 157% increase over WT-*Ph1*prol. Furthermore, the activities for the *Ph1*prol mutants against *p-*nitrophenyl soman (0.7, 1.0, and 0.9 U/mg for Y301C/K342N-, E127G/E252D-, and E36V-*Ph1*prol, resp.) compare favorably to the improved soman analog activities reported for the *P. furiosus *prolidase mutants (0.86, 1.02, and 1.7 U/mgfor G39E-, R19G/K71E/S229T-, and R19G/G39E/K71E/S229T-*Pf*prol, resp.) [20]. When looking at the substrate specificity of the WT-*Ph1*prol and variants with proline dipeptides, it was noticed that there was a shift in preference from more hydrophobic to less hydrophobic amino acids among the mutants. This is also seen with the OP nerve agents, where there is a shift in substrate specificity from DFP to the soman analog. The WT-*Ph1*prol prefers DFP as a substrate over the soman analog, while the *Ph1*prol variants show decreased activity with DFP and increased activity with the soman analog. 

## 4. Conclusion

Current biodecontamination formulations for degradation of OP nerve agents that incorporate *Alteromonas* prolidases (OPAA) and PTE have limitations when used in the field [12]. Long-term stability of the enzyme is not attainable in a formulation mixture that includes other solvents and denaturing solutions, and the need to add excess metal to reach maximum activity poses further complications for an enzyme-based detoxification system. A highly active enzyme that is stable over the long term and requires very little metal addition would be best suited for this application. The wild type and mutant prolidases characterized from *P. horikoshii* show promising enzymatic properties that make them potential candidates for future optimization studies for OP nerve agent degradation. Compared to *Pf*prol, *Ph1*prol and the four *Ph1*prol mutants show higher activity, higher affinity for the substrate, and significantly lower metal requirement for catalysis. Two of the variants, Y301C/K342N- and E127G/E252D-*Ph1*prol are thermostable for nearly three times as long as *Pf*prol and double the time of *Ph1*prol. A195T/G306S-*Ph1*prol has 808% of the DFP activity compared to wild type *P. furiosus *prolidase and is superior to any of the improved *P. furiosus *prolidase mutants [21]. Furthermore, Y301C/K342N-, E127G/E252D-, and E36V-*Ph1*prol all have improved activities against *p-*nitrophenyl soman relative to WT-*Ph1*prol and also compare favorably to the best performing *P. furiosus *prolidase mutants [20]. The *Ph1*prol variants have the potential to significantly improve upon current biodecontamination strategies. Their increased thermostability and pot life and activities against OP nerve agent analogs warrant further study into large-scale production and purification of these prolidases.

## Figures and Tables

**Table 1:** Substrate specificity of recombinant wild type and variant *P. horikoshii* prolidases with different proline dipeptides and a single-proline tripeptide.

Substrate	Relative Activity (%) of WT-*Ph1*prol-specific activity				
	WT- *Ph1*prol	A195T/ G306S- *Ph1*prol	Y301C/ K342N- *Ph1*prol	E127G/ E252D- *Ph1*prol	E36V- *Ph1*prol
Val-Pro	100	33	36	46	56
	(4,602)				
Met-Pro	100	143	33	117	77
	(2,809)				
Phe-Pro	100	116	53	53	68
	(2,199)				
Leu-Pro	100	116	36	90	65
	(2,132)				
Ala-Pro	100	219	183	324	556
	(1,452)				
Gly-Pro	100	94	52	107	444
	(369)				
Pro-Ala	100	137	33	75	332
	(14)				
Val-Leu-Pro	100	0	88	0	0
	(2)				

Prolidase assays were performed at 100°C and contained 0.2 mM CoCl_2_ and 4 mM of each substrate. One hundred percent specific activity is reported for WT-*Ph1*prol and correlates to U/mg in parentheses below the 100% relative activity.

**Table 2:** Kinetic parameters of wild type *Pyrococcus horikoshii* prolidase, *Ph1*prol, and prolidase variants with Leu-Pro at 70°C.

Prolidase	* K_m _* (mM)	*V*_max⁡_ (*μ*mol/min/mg)	*k*_cat_ (s^−1^)	*k*_cat_ */K_m _* (mM^−1^s^−1^)
WT-*Ph1*prol	0.92 ± 0.16	809 ± 55	1079	1172
A195T/G306S	0.81 ± 0.26	1245 ± 149	1660	2049
Y301C/K342N	2.92 ± 0.40	2146 ± 177	2861	980
E127G/E252D	0.98 ± 0.24	1119 ± 101	1492	1522
E36V	1.6 ± 0.37	1597 ± 174	2129	1331

Enzyme assays were done using a range of Leu-Pro concentrations (0.25–12 mM). Enzyme kinetics were calculated using nonlinear regression (curve fit) and analyzed using the Michaelis-Menten equation utilizing software from Prism 5 (GraphPad La Jolla, CA). All *Ph1*prol assays contained 0.2 mM CoCl_2_.

**Table 3:** Transition temperature of wild type *Pyrococcus horikoshii* prolidase, *Ph1*prol, and prolidase variants at pH 7.0.

Prolidase	*T_m _*(°C)
WT-*Ph1*prol	114.3 ± 1.1
A195T/G306S^1^	114.4 ± 0.1
Y301C/K342N	113.8 ± 0.6
E127G/E252D	112.2 ± 0.5
E36V	114.9 ± 1.3

*T_m_* is the transition temperatures obtained from the analysis of DSC by using the two-state model. The *T_m_* values are the mean of three independent measurements. ^1^The *T_m_* value is from two independent measurements.
